# Serum soluble alpha-klotho klotho and cognitive functioning in older adults aged 60 and 79: an analysis of cross-sectional data of the National Health and Nutrition Examination Survey 2011 to 2014

**DOI:** 10.1186/s12877-024-04661-7

**Published:** 2024-03-11

**Authors:** Song Ge, Fanghong Dong, Chong Tian, Chih-Hsiang Yang, Minhui Liu, Jingkai Wei

**Affiliations:** 1 College of Sciences and Technology, University of Houston-Downtown, Houston, TX US; 2 Department of Psychiatry, Washington University in St. Louis, St. Louis, Missiouri United States of America; 3 School of Nursing, Tongji Medical College, Huazhong University of Science and Technology, Wuhan, Hubei Province China; 4 Department of Exercise Science, Arnold School of Public Health, University of South Carolina, Columbia, SC US; 5 School of Nursing, Ningxia Medical University, No. 1160, Shengli Street, Xingqing District, 410013 Yinchuan, Ningxia China; 6 Department of Epidemiology and Biostatistics, Arnold School of Public Health, University of South Carolina, Columbia, SC US

**Keywords:** Klotho, Cognitive function, Older adults, NHANES, Alpha-klotho

## Abstract

**Objectives:**

Klotho, consisting of membrane klotho and soluble alpha-klotho, is found to be associated with better cognitive outcomes in small samples of the aged population. We aimed to examine the association of serum soluble alpha-klotho with cognitive functioning among older adults using a nationally representative sample of U.S. older adults.

**Method:**

A total of 2,173 U.S. older adults aged 60–79 years in the National Health and Nutrition Examination Survey from 2011 to 2014 were included in this cross-sectional analysis. Serum soluble alpha-klotho was measured in the laboratory and analyzed with an ELISA kit. Cognitive function was measured using the Consortium to Establish a Registry for Alzheimer’s Disease Word Learning subtest (CERAD-WL) immediate and delayed memory, the Animal fluency test (AFT), and the Digit Symbol Substitution Test (DSST). Test-specific and global cognition z-scores were calculated based on sample means and standard deviations. Multivariable linear regression models were applied to examine the association of quartiles and continuous value of serum soluble alpha-klotho with test-specific and global cognition z-scores. Subgroup analysis was conducted by sex. The following covariates were included in the analysis- age, sex, race/ethnicity, education, depressive symptoms, smoking status, body mass index (BMI), physical activity, stroke, prevalent coronary heart disease, total cholesterol, and systolic blood pressure. All the information was self-reported or obtained from health exams.

**Results:**

Serum soluble alpha-klotho level in the lowest quartile was associated with lower z-scores for DSST (beta [β] =-0.13, 95% confidence interval [CI]: -0.25, -0.01). For subgroup analysis, serum soluble alpha-klotho level in the lowest quartile was associated with lower z-scores for DSST (β=-0.16, 95% CI: -0.32, -0.003) and global cognition (β=-0.14, 95% CI: -0.28, -0.01) among female participants. No association was found between continuous serum soluble alpha-klotho and cognitive functioning among the participants.

**Conclusions:**

Lower serum soluble alpha-klotho quartile was associated with poorer cognitive functioning among older women. Future studies are expected to examine the longitudinal association between klotho levels and cognitive outcomes.

**Supplementary Information:**

The online version contains supplementary material available at 10.1186/s12877-024-04661-7.

## Introduction

Older age is associated with a higher risk of cognitive decline [1]. Cognitive decline is associated with increased mortality, disability, and loss of independence in older adults [2]. As such, there is a growing need for interventions targeting at slowing the progression of cognitive decline. Performance in cognitive tests is an important measurement of cognitive functioning among older adults. Risk factors of cognitive decline may be identified by looking at their relationships with cognitive functioning as manifested in the cognitive tests.

Klotho, a transmembrane protein encoded by the KL gene, which is predominantly expressed in the kidney and the brain, could be related to cognitive aging [3, 4]. There are two forms of klotho, membrane klotho and soluble klotho, which exert different functions. By forming a complex with fibroblast growth factor (FGF) receptors, membrane klotho acts as an obligatory co-receptor for a bone-derived hormone that promotes phosphate excretion into urine [5]. Soluble alpha-klotho functions like a hormone and exerts systematic effects on multiple organs in the body. Serum klotho level declines as human ages [6] and klotho is considered to have anti-aging properties through its systematic effects on the body and the brain [7]. Klotho may therefore play an important role in the brain and affect cognitive function. People with cognitive decline are found to have a lower klotho level in their cerebrospinal fluid (CSF) compared to those who are cognitively normal [8].

Only a few studies have been conducted to examine the associations between klotho level and cognitive outcomes in human populations and indicated that low blood klotho is associated with poorer cognitive outcomes [9–12]. However, all these studies were based on selected samples of populations with small sample sizes. While klotho has been considered a potential key to healthy brain aging [4], it is desirable to examine the association between klotho and cognitive function in a more representative sample of the aged population, so that relationship between klotho and cognition may be better elucidated. Compared with elderly men, elderly women were more likely to experience a faster cognitive decline partly due to excessive sensitivity to hormonal changes in the brain and different brain structures (13–16). Thus, the cognitive impact of serum klotho, which functions like hormones in the body [7, 13] may affect women and men differently. Therefore, we aimed to examine whether the associations between klotho and cognitive function differ between men and women.

In this study, we took advantage of the National Health and Nutrition Examination Survey (NHANES) 2011 to 2014 [14, 15] to examine the association between serum soluble alpha-klotho and cognitive functioning in a nationally representative sample of older adults in the U.S., with additional analysis on the associations among men and women, respectively.

## Methods

### The NHANES study design

The NHANES is a continuous cross-sectional survey of civilian, non-institutionalized U.S. adults and children conducted bi-annually by the National Center for Health Statistics of the Centers for Disease Control and Prevention [14]. For each cycle, participants across the country are recruited from a selection of census blocks or clusters of census block area segments, which makes the NHANES a nationally representative sample of the U.S [14, 15].. The detailed sampling method adopted has been published elsewhere [16]. Face-to-face interviews and health examinations are used to assess participants’ sociodemographic, health, and nutritional status.

For this analysis, we included participants in the NHANES 2011 to 2014 with available information on serum klotho levels and cognitive function performance. A total of 19,931 individuals participated in the NHANES. We excluded individuals aged < 60 (*n* = 16,299) or had missing data on cognitive tests (*n* = 444), serum klotho (serum klotho was only collected in individuals aged 40 to 79) (*n* = 1,014), or education (*n* = 1). Finally, a total of 2,173 participants aged 60 and 79 were included in the analysis.

### Ethical considerations

The National Center for Health Statistics Research Ethics Review Board approved the NHANES. This study was granted an exemption by the University of Houston-Downtown Committee for the Protection of Human Subjects because only de-identified, publicly available data were used.

### Measures

#### Independent variable: quartile of serum soluble alpha-klotho

Serum soluble alpha-klotho, specifically soluble alpha-klotho, was measured in the laboratory and analyzed with an ELISA kit made by IBL International, Japan [17]. The intra-assay precision measured on two human samples and two recombinant Klotho samples showed coefficients of variation of 2.3% and 3.3% for the human samples and 3.2% and 3.9% for the recombinant samples [17]. The sensitivity of the assay was 6 pg/mL. The levels of serum klotho among 2,173 participants ranged from 206.0 to 3,456 pg/mL. We used serum soluble alpha-klotho quartile to be consistent with many published NHANES biomarker studies [18–20]. For analysis, serum klotho was categorized into four quartiles: <657.8pg/mL, 657.8 -797.7 pg/mL, 797.8-958.2 pg/mL, and ≥ 958.3 pg/mL.

#### Dependent variable: cognitive functioning

Three cognitive tests, including the Consortium to Establish a Registry for Alzheimer’s Disease Word Learning subtest (CERAD-WL), the Animal Fluency test (AFT), and the Digit Symbol Substitution Test (DSST) were used to assess participants’ cognitive function.

The CERAD-WL evaluated participants’ ability to acquire new verbal knowledge through immediate memory and delayed memory [21]. It consisted of a delayed recall after three consecutive learning trials. For each learning trial, participants were asked to read out aloud ten random words presented in large, bolded letters on a computer monitor, one at a time. Participants were instructed to remember and recall as many words as they could right after the words were presented. The placement of the ten words was altered in each of the three trials, and each trial, therefore, had a possible maximum score of ten. The total score of three trials was a participant’s immediate memory score and ranged between 0 and 30. The delayed recall test, which was conducted after the other two cognitive tests, required individuals to recall as many words from the same ten-word list as they could. The number of correct words recalled was a participant’s delayed memory score and ranged between 0 and 10.

The AFT assessed participants’ language fluency and executive function [22]. Participants were required to name as many animals as they could in one minute and received one score for each animal named.

The DSST assessed participants’ processing speed, sustained attention, and working memory [23]. The test was administered utilizing a paper form with a top-mounted key that contained nine numbers with paired symbols. The 133 boxes next to the numbers contained corresponding symbols, and participants had two minutes to copy the symptoms to the 133 boxes. The total number of right matches determined the score [24], and the possible score range of the DSST was between 0 and 133 [25].

#### Covariates

To account for potential confounding between serum soluble alpha-klotho and cognitive function, we reviewed relevant literature and included the following covariates for the analysis: age (years), sex (male or female), race/ethnicity (Mexican Americans, other Hispanics, non-Hispanic White, or non-Hispanic Black), education (below high school, high school graduate, or some college or above), depressive symptoms, smoking status (never, former, or current smokers), body mass index (BMI) (< 18.5 kg/m^2^, 18.5–24.9 kg/m^2^, 25-29.9 kg/m^2^, or ≥ 30 kg/m^2^), physical activity (hour of moderate to intense exercise per day), stroke (yes or no), prevalent coronary heart disease (CHD) (yes or no), total cholesterol (mg/dL), and systolic blood pressure (mmHg). The Patient Health Questionnaire (PHQ-9) was used to measure depressive symptoms [26]. All the information was self-reported or obtained from health exams.

### Statistical analysis

Using sample means and standard deviations of the cognitive test scores, test-specific z-scores were calculated for the CERAD-WL immediate memory, the CERAD-WL delayed memory, the AFT, and the DSST, respectively. The global cognition z-score was then calculated by means and standard deviations of all test-specific z-scores. The characteristics of the study population, stratified by quartile of serum soluble alpha-klotho, were presented and linear trend analysis was conducted. For the linear trend analysis, we used unadjusted linear/logistic regression models, with quartiles of serum soluble alpha-klotho as independent variables, and continuous/binary variables as dependent variables. ANOVAs were used to examine the difference in the cognitive tests between quartiles of serum soluble alpha-klotho. Multivariable regression models were constructed between quartiles of serum soluble alpha-klotho and continuous serum soluble alpha-klotho and test-specific and global z-scores with adjustment of all the covariates listed above. We tested the interaction between sex and serum klotho by including an interaction term of serum klotho and sex in the models. An interaction existed if the *P*-value for the interaction terms was smaller than 0.05. Women and men were fit separately to account for sex differences in the effects of klotho on cognitive functioning. The analyses were weighted to account for survey non-response and the stratified, multistage probability sampling method of the NHANES. For subsamples in 2011–2012 and 2013–2014, the full sample 2-year mobile examination center exam weight was used, and the weights were recalculated and divided by two after the combination of NHANES 2011–2012 with 2013–2014 [27]. We considered a 95% confidence interval (CI) not including zero as statistically significant. All analyses were performed using SAS 9.4 (Cary, NC).

## Results

The characteristics of the excluded participants (*n* = 641) were in Supplemental Table 1. Compared with the included participants, the excluded participants were more likely to be non-Hispanic Black (17.0%) and other race/ethnicity (11.1%) and had less physical activity per day (0.6 h).

The characteristics of the study population, stratified by serum soluble alpha-klotho quartile, were presented in Table 1. Of the 2,173 participants, 1,017 were from the 2011–2012 cycle and 1,156 from the 2013–2014 cycle, which represented a population size of 40,972,624. They had a mean age of 67.4 years. Roughly 53% of the participants were female. The majority, 78.6%, were of non-Hispanic White ethnicity. Around 62% had achieved at least some college education. Additionally, 39.7% had a BMI of ≥ 30 kg/m², while their mean daily physical activity amounted to 0.9 h. A small percentage of the participants had stroke (5.7%), and 8.0% of the participants had CHD. The participants had a mean total cholesterol of 194 mg/dL and a mean systolic blood pressure of 130.4 mmHg. Their mean CERAD W-L delayed memory, CERAD W-L immediate memory, AFT, and DSST scores were 6.4 ± 0.1, 20.0 ± 0.2, 18.5 ± 0.2, and 54.5 ± 0.6, respectively. Compared with participants in the highest klotho category, those in the lower serum klotho category were older (*P*-value for linear trend = 0.01), more likely to be male (*P*-value for linear trend = 0.03), more likely to have prevalent CHD (*P*-value for linear trend = 0.03), and had a lower DSST score (*P*-value for linear trend = 0.02).

**Table 1:** Weighted characteristics of the participants by serum soluble alpha-klotho quartile^a^

	Quartile 1 < 657.8pg/mL (*n* = 537)	Quartile 2 657.8 -797.7pg/mL (*n* = 510)	Quartile 3 797.8-958.2 pg/mL (*n* = 525)	Quartile 4 ≥ 958.3 pg/mL (*n* = 601)	Total (*n* = 2,173)	*P*-value for linear trend
Weighted number of participants	10,242,822	10,232,107	10,227,089	10,270,606	40,972,624	-
Age, years	68.1 (0.3)	67.5 (0.3)	67.1 (0.4)	67.1 (0.2)	67.4 (0.2)	**0.01**
Female, n (%)	271 (52.1)	242 (49.9)	269 (52.4)	337 (57.9)	1,119 (53.1)	**0.03**
Race/ethnicity, n (%)						0.55
Mexican Americans	53 (3.6)	54 (3.5)	57 (3.9)	67 (4.5)	231 (3.9)	
Other Hispanics	44 (2.9)	58 (3.6)	68 (4.4)	79 (4.9)	249 (4.0)	
Non-Hispanic Whites	253 (79.4)	232 (80.9)	227 (79.7)	218 (74.5)	930 (78.6)	
Non-Hispanic Blacks	134 (8.2)	114 (7.3)	105 (6.5)	175 (10.8)	528 (8.2)	
Other	53 (5.8)	52 (4.7)	68 (5.5)	62 (5.4)	235 (5.4)	
Education, n (%)						0.45
Below high school	153 (17.4)	151 (17.8)	132 (13.1)	155 (16.1)	591 (16.1)	
High school graduate	125 (23.9)	106 (19.5)	114 (19.0)	146 (23.8)	491 (21.6)	
Some college or above	259 (58.7)	253 (62.7)	279 (68.0)	300 (60.1)	1,091 (62.4)	
CES-D score	2.9 (0.2)	2.8 (0.2)	2.9 (0.3)	2.8 (0.2)	2.8 (0.1)	0.81
Smoking status, n (%)						0.59
Never	249 (48.1)	221 (46.6)	262 (51.2)	327 (50.1)	1,059 (49.0)	
Former	211 (39.0)	208 (42.2)	191 (37.7)	193 (35.6)	803 (38.6)	
Current	77 (12.9)	80 (11.2)	72 (11.2)	81 (14.4)	310 (12.4)	
Body mass index, n (%)						0.15
<18.5 kg/m^2^	6 (0.5)	6 (1.2)	5 (1.2)	5 (1.5)	22 (1.1)	
18.5–24.9 kg/m^2^	120 (19.4)	114 (22.0)	125 (22.9)	152 (25.5)	511 (22.5)	
25.0-29.9 kg/m^2^	187 (38.7)	191 (39.0)	183 (35.1)	203 (34.2)	764 (36.7)	
≥30 kg/m^2^	217 (41.3)	194 (37.8)	206 (40.8)	238 (38.8)	855 (39.7)	
Physical activity, hours/week	1.1 (0.1)	1.3 (0.1)	1.4 (0.1)	1.3 (0.1)	1.3 (0.1)	0.28
Stroke, n (%)	42 (6.8)	34 (6.2)	31 (5.9)	31 (4.1)	138 (5.7)	0.13
Prevalent coronary heart disease, n (%)	50 (9.2)	47 (9.3)	42 (8.8)	25 (4.8)	164 (8.0)	**0.03**
Total cholesterol, mg/dL	190.9 (3.7)	193.9 (3.5)	195.9 (2.0)	195.7 (2.6)	194.1 (1.3)	0.12
Systolic blood pressure, mmHg	130.4 (1.3)	130.7 (1.0)	129.7 (1.3)	131.0 (1.1)	130.4 (0.5)	0.89
CERAD W-L immediate recall	19.9 (0.3)	19.8 (0.3)	20.1 (0.3)	20.3 (0.3)	20.0 (0.2)	0.15
CERAD W-L delayed recall	6.3 (0.2)	6.4 (0.2)	6.6 (0.2)	6.5 (0.1)	6.4 (0.1)	0.10
Animal fluency test	18.2 (0.3)	18.6 (0.3)	19.0 (0.4)	18.2 (0.3)	18.5 (0.2)	0.72
Digit Symbol Substitution Test	51.8 (0.9)	54.7 (1.0)	56.1 (1.1)	54.8 (1.0)	54.4 (0.6)	**0.02**

a. Data was presented as mean (standard deviation) for continuous variables and n (%) for categorical variables

**Fig. 1:**
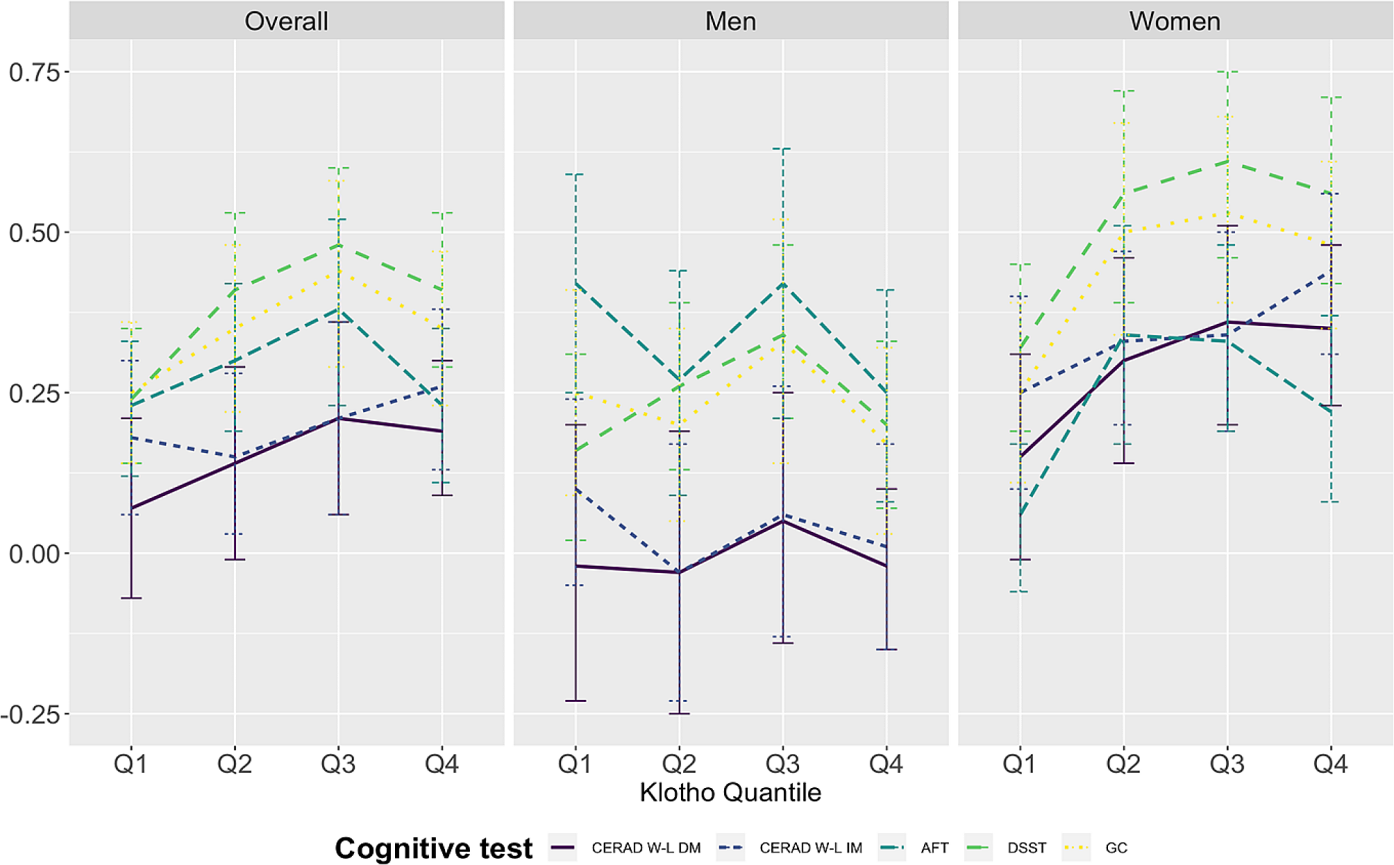
Cognitive z-scores and 95% confidence intervals by serum soluble alpha-klotho quartile. *Notes.* Quartile (Q) 1: <657.8pg/mL; Q2: 657.8 -797.8 pg/mL; Q3: 797.8-958.3 pg/mL; Q4: ≥ 958.3 pg/mL; CEARD W-L DM: CREAD W-L delayed recall memory; CEARD W-L IM: CREAD W-L immediate recall memory; AFT: Animal fluency test; DSST: Digit Symbol Substitution Test; GC: Global cognition. The error bar presents 95% confidence intervals of cognition z-score at each quartile of serum soluble alpha-klotho

With a higher quartile of serum soluble alpha-klotho, the global cognition z-score showed an increasing trend with higher quartiles of serum klotho. Stratified by sex, increasing linear trends were found for z-scores for DSST, the CERAD-WL immediate memory, the CERAD-WL delayed memory, and global cognition z-scores among female participants. No linear trend was found between serum soluble alpha-klotho and cognition z-scores among male participants (Fig. 1).

**Table 2:** Cognitive z-scores by quartile of serum soluble alpha-klotho

		< 657.8pg/mL	657.8 -797.8 pg/mL	797.8-958.3 pg/mL	≥ 958.3 pg/mL	*P*-value for ANOVA
Digit Symbol Test	Overall	0.24 (0.14, 0.35)	0.41 (0.29, 0.53)	0.48 (0.36, 0.60)	0.41 (0.29, 0.53)	**0.0003**
	Men	0.16 (0.02, 0.31)	0.26 (0.13, 0.39)	0.34 (0.21, 0.48)	0.20 (0.07, 0.33)	**0.003**
	Women	0.32 (0.19, 0.45)	0.56 (0.39, 0.72)	0.61 (0.46, 0.75)	0.56 (0.42, 0.71)	**< 0.0001**
CERAD W-L delayed recall	Overall	0.07 (-0.07, 0.21)	0.14 (-0.01, 0.29)	0.21 (0.06, 0.36)	0.19 (0.09, 0.30)	0.07
	Men	-0.02 (-0.23, 0.20)	-0.03 (-0.25, 0.19)	0.05 (-0.14, 0.25)	-0.02 (-0.15, 0.10)	0.44
	Women	0.15 (-0.01, 0.31)	0.30 (0.14, 0.46)	0.36 (0.20, 0.51)	0.35 (0.23, 0.48)	**0.001**
CERAD W-L immediate recall	Overall	0.18 (0.06, 0.30)	0.15 (0.03, 0.28)	0.21 (0.06, 0.36)	0.26 (0.13, 0.38)	0.29
	Men	0.10 (-0.05, 0.24)	-0.03 (-0.23, 0.17)	0.06 (-0.13, 0.26)	0.01 (-0.15, 0.17)	0.09
	Women	0.25 (0.10, 0.40)	0.33 (0.20, 0.47)	0.34 (0.19, 0.50)	0.44 (0.31, 0.56)	**0.01**
Animal fluency test	Overall	0.23 (0.12, 0.33)	0.30 (0.19, 0.42)	0.38 (0.23, 0.52)	0.23 (0.11, 0.35)	**0.05**
	Men	0.42 (0.25, 0.59)	0.27 (0.09, 0.44)	0.42 (0.21, 0.63)	0.25 (0.08, 0.41)	**0.004**
	Women	0.06 (-0.06, 0.17)	0.34 (0.17, 0.51)	0.33 (0.19, 0.48)	0.22 (0.08, 0.37)	**< 0.0001**
Global cognition	Overall	0.25 (0.14, 0.36)	0.35 (0.22, 0.48)	0.44 (0.29, 0.58)	0.35 (0.23, 0.47)	**0.02**
	Men	0.25 (0.09, 0.41)	0.20 (0.05, 0.35)	0.33 (0.14, 0.52)	0.17 (0.03, 0.32)	**0.04**
	Women	0.25 (0.11, 0.39)	0.50 (0.34, 0.67)	0.53 (0.39, 0.68)	0.48 (0.35, 0.61)	**< 0.0001**

ANOVA showed that there were statistically significant differences in the DSST, the CERAD W-L delayed recall, the CERAD W-L immediate recall, the AFT, and global cognition z scores between different serum soluble alpha-klotho quartiles among the participants (Table 2).

Multivariable linear regression showed that compared with participants in the highest quartile of serum soluble alpha-klotho, participants in the lowest quartile had 0.13 standard deviations lower for DSST (β=-0.13, 95% CI -0.25, -0.01). No interaction was found between serum klotho and sex. For subgroup analysis, compared with female participants in the highest quartile of serum soluble alpha-klotho, female participants in the lowest quartile had 0.16 standard deviations lower for DSST (β=-0.16, 95% CI -0.32, -0.003). Moreover, compared with female participants in the highest quartile of serum soluble alpha-klotho, female participants in the lowest quartile had a 0.14 standard deviations lower global cognition (β=-0.14, 95% CI -0.28, -0.01). No association was found between serum soluble alpha-klotho quartile and cognitive functioning among male participants. No association was found between continuous serum soluble alpha-klotho and cognitive functioning among the participants (Table 3).

**Table 3:** The independent associations of serum soluble alpha-klotho quartile and serum soluble alpha-klotho (continuous) with test-specific and global cognition z-scores^a^

		Quartile 1 < 657.8pg/mL (*n* = 537)	Quartile 2 657.8 -797.7pg/mL (*n* = 510)	Quartile 3 797.8-958.2 pg/mL (*n* = 525)	Quartile 4 ≥ 958.3 pg/mL (*n* = 601)	Continuous
		β (95% confidence interval)	β (95% confidence interval)	β (95% confidence interval)	β (95% confidence interval)	β (95% confidence interval)
CERAD W-L immediate recall	Overall	-0.02 (-0.13, 0.10)	-0.07 (-0.21, 0.06)	-0.07 (-0.22, 0.08)	Reference	0.0001 (-0.00003, 0.0003)
	Men	0.09 (-0.07, 0.26)	-0.06 (-0.28, 0.16)	0.03 (-0.16, 0.23)	Reference	0.0001 (-0.0001, 0.0003)
	Women	-0.11 (-0.29, 0.06)	-0.08 (-0.20, 0.05)	-0.15 (-0.32, 0.02)	Reference	0.0001 (-0.00004, 0.0003)
CERAD W-L delayed recall	Overall	-0.08 (-0.23, 0.07)	-0.04 (-0.21, 0.13)	0.01 (-0.14, 0.15)	Reference	0.0001 (-0.0001, 0.0003)
	Men	0.003 (-0.25, 0.26)	-0.02 (-0.30, 0.25)	0.07 (-0.13, 0.28)	Reference	-0.00001 (-0.0004, 0.0004)
	Women	-0.16 (-0.35, 0.02)	-0.05 (-0.22, 0.12)	-0.05 (-0.22, 0.13)	Reference	0.0002 (-0.00004, 0.0004)
Digit Symbol Substitution Test	Overall	**-0.13** **(-0.25, -0.01)**	-0.01 (-0.16, 0.14)	0.02 (-0.09, 0.12)	Reference	0.00003 (-0.0001, 0.0002)
	Men	-0.08 (-0.24, 0.07)	0.01 (-0.17, 0.19)	0.10 (-0.06, 0.26)	Reference	-0.0001 (-0.0003, 0.0001)
	Women	**-0.16** **(-0.32, -0.003)**	-0.02 (-0.18, 0.14)	-0.05 (-0.18, 0.08)	Reference	0.0001 (-0.0001, 0.0003)
Animal fluency test	Overall	0.03 (-0.08, 0.14)	0.05 (-0.10, 0.19)	0.07 (-0.09, 0.22)	Reference	-0.0001 (-0.0003, 0.0001)
	Men	0.14 (-0.05, 0.33)	-0.03 (-0.26, 0.21)	0.09 (-0.13, 0.30)	Reference	-0.0001 (-0.0004, 0.0002)
	Women	-0.08 (-0.24, 0.07)	0.11 (-0.06, 0.27)	0.04 (-0.13, 0.21)	Reference	-0.00003 (-0.0003, 0.0002)
Global cognition	Overall	-0.05 (-0.14, 0.05)	0.003 (-0.14, 0.15)	0.03 (-0.10, 0.16)	Reference	0.00002 (-0.0001, 0.0002)
	Men	0.05 (-0.12, 0.23)	-0.01 (-0.23, 0.20)	0.10 (-0.09, 0.28)	Reference	-0.00004 (-0.0003, 0.0002)
	Women	**-0.14** **(-0.28, -0.01)**	0.02 (-0.12, 0.16)	-0.03 (-0.17, 0.10)	Reference	0.0001 (-0.0001, 0.0003)

a. The models were adjusted for age (years), sex (male or female), race/ethnicity (Mexican Americans, other Hispanics, non-Hispanic White, or non-Hispanic Black), education (below high school, high school graduate, or some college or above), depressive symptoms, smoking status (never, former, or current smokers), BMI (< 18.5 kg/m2, 18.5–24.9 kg/m2, 25-29.9 kg/m2, or ≥ 30 kg/m2), physical activity (hour of moderate to intense exercise per day), stroke (yes or no), prevalent coronary heart disease (CHD) (yes or no), total cholesterol (mg/dL), and systolic blood pressure (mmHg)

## Discussion

In this nationally representative sample of U.S. older adults, lower serum soluble alpha-klotho was associated with worse executive function, processing speed, and global cognition, particularly in older women. This relationship is independent of the covariates.

Previous studies have examined the associations of klotho levels with cognitive outcomes. In a secondary analysis using a randomized controlled trial of 103 nursing home residents in Spain, lower klotho level is associated with lower processing speed measured with the Coding Wechsler Adult Intelligence Scale [9]. In the InCHIANTI Study, among 855 older adults aged 55 and above in Italy, no statistically significant association is found between serum klotho level and cognitive function measured with Trails A and B or Mini-Mental State Examination (MMSE) measured after three years, while higher serum klotho level is associated with lower risk of 3-year cognitive decline measured with MMSE [10]. Kundu et al. found in 94 adults aged between 39 and 83 + that α-klotho levels in serum and cerebrospinal fluid are predictive of higher scores on MMSE and lower scores on Clinical Dementia Rating (CDR) [11]. In another study by Kundu et al., a positive relationship between apoE and alpha-klotho was identified in the amygdala of aged male Rhesus macaques [28]. In addition, Brombo et al. found that a lower level of klotho is associated with a higher prevalence of vascular dementia among older adults [12]. All these studies suggested that a low level of klotho is associated with unfavorable cognitive outcomes, no matter whether they used cross-sectional or longitudinal designs. This is consistent with our findings. However, most of these studies were based on small and selective samples while we took advantage of a relatively diverse and large sample and found similar results, which added stronger evidence on the negative association between klotho level and cognitive outcomes in humans.

The mechanisms that account for the association between klotho and cognitive functioning are complicated. Klotho has been shown to alter the shape of synapses in the hippocampus and cortex in humans, regions of the brain important for learning and memory [29]. Mice with klotho deficiency showed brain atrophy [30] learning difficulties [31] a reduction in hippocampal synapses, axonal transport problems, hippocampus neurodegeneration [32] and demyelination [32]. In contrast, mice with genetically engineered enhanced expression of klotho showed better cognition across life span than general mice [33]. Specifically, elevated klotho improved spatial learning and memory in human amyloid precursor protein (hAPP) transgenic mice by preventing the loss of NMDA receptor (NMDAR) subunits in the hippocampus [34]. In addition, the choroid plexus, a structure that expresses klotho and creates CSF, and klotho are involved in calcium transfer to the central nervous system [35]. The movement of calcium ions across the membrane of the granule pyramidal cells is important for long-term potentiation and cognition [36]. Future studies should explore the mechanisms of sex difference in the cognitive effects of klotho and explain why the mechanisms mentioned above were not significant in older men.

With the large and representative sample, we were able to conduct subgroup analysis based on sex, and we found that the associations were more prominent among older women. The mechanisms that account for different associations found between older men and older women are not determined yet, although some biological processes can be used for potential explanation. Serum klotho functions as a hormone and exhibits systematic effects in the body [7, 13]. Compared with older men, older women have increased sensitivity to hormonal changes in the brain, increased tau pathology burden [37] and have different brain structures, genetic components, and psychosocial factors [38–41]. Older women are also more likely to experience faster cognitive decline than older men [38]. These sex-related differences may account for the difference in the cognitive effects of klotho. As older women are more likely to experience delayed cognitive decline recognition and a faster cognitive decline trajectory [38], our study targets a problem of high public health importance in a vulnerable population. In older women, serum klotho may be used as a biomarker for risk stratification of cognitive impairment. A better understanding of the multiple mechanisms where klotho exerts its cognitive protective roles in older women may enable the identification of cognitive impairment and timely prevention and treatment.

Our study has important public health implications as it showed that older women with the lowest quartile of serum klotho were likely to have poorer cognitive functioning. This finding can help clinicians and public health professionals identify the subgroup of the population with a relatively higher risk of cognitive impairment and dementia so that timely treatment and prevention can be initiated to improve cognitive function among these individuals. This will alleviate the public health burden brought by cognitive aging.

Our study is subject to several limitations. Firstly, our study is cross-sectional; thus, no temporal relationship can be established. The possibility of klotho being reduced with poor cognitive function through suboptimal profiles of lifestyle or psychosocial factors cannot be excluded. Moreover, we only had three cognitive tests, which may not be able to assess cognitive function comprehensively. In addition, information on serum klotho is lacking among older adults ≥ 80 years old, so the associations of serum klotho with cognitive functioning could not be examined among people of this age group. Third, the analysis where serum soluble alpha-klotho was a continuous variable did not yield statistically significant findings as opposed to the one where serum soluble alpha-klotho was a categorical variable. This indicates that our findings may not be robust and need validation from future studies. Despite these limitations, our study was based on a large and nationally representative sample of older adults, with high-quality data of serum soluble alpha-klotho and cognitive tests. Our study is also the first study that identified a sex-specific cognitive effect of serum soluble alpha-klotho and added strong evidence to the negative association between klotho and cognitive functioning among older women, thus making a unique contribution to the literature. Future studies are needed to examine the longitudinal association between klotho concentration in the brain and cognitive functioning in humans.

In conclusion, lower serum soluble alpha-klotho is associated with poorer executive function and processing speed, as well as global cognition in older women. Future studies are expected to examine the longitudinal associations between klotho levels and cognitive outcomes.

### Electronic supplementary material

Below is the link to the electronic supplementary material.

Supplementary Material 1

## Data Availability

The data that support the findings of this study are openly available on the NHANES website and can be accessed at https://wwwn.cdc.gov/nchs/nhanes/Default.aspx.
