# Supplementary material for: Serum soluble alpha-klotho klotho and cognitive functioning in older adults aged 60 and 79: an analysis of cross-sectional data of the National Health and Nutrition Examination Survey 2011 to 2014
Source: BMC Geriatr. 2024 Mar 11;24:245. doi: 10.1186/s12877-024-04661-7 (PMC10929106; doi:10.1186/s12877-024-04661-7)
Supplement: Supplementary file 1 — Supplementary Material 1 [file 12877_2024_4661_MOESM1_ESM.docx]

Supplemental Table 1. The characteristics of included participants and excluded participants due to missing data

|  | Excluded  (n=641) | Included  (n=2,173) |
| --- | --- | --- |
| Weighted n | 8,405,952 | 40,972,624 |
| Age, year | 67.8 (0.4) | 67.4 (0.2) |
| Female, n (%) | 313 (55.0) | 1,119 (53.1) |
| Race/ethnicity, n (%) |  |  |
| Mexican Americans | 71 (6.1) | 231 (3.9) |
| Other Hispanics | 63 (5.1) | 249 (4.0) |
| Non-Hispanic Whites | **174 (60.8)** | **930 (78.6)** |
| Non-Hispanic Blacks | **226 (17.0)** | **528 (8.2)** |
| Other | **107 (11.1)** | **235 (5.4)** |
| Education, n (%) |  |  |
| Below high school | 227 (23.9) | 591 (16.1) |
| High school graduate | 146 (21.4) | 491 (21.6) |
| Some college or above | 265 (54.7) | 1,091 (62.4) |
| Smoking, n (%) |  |  |
| Never | 307 (46.7) | 1,059 (49.0) |
| Former | 220 (36.9) | 803 (38.6) |
| Current | 113 (16.4) | 310 (12.4) |
| Body mass index, kg/m^2^ |  |  |
| <18.5 | 22 (3.0) | 22 (1.1) |
| 18.5-24.9 | 170 (28.2) | 511 (22.5) |
| 25.0-29.9 | 184 (28.5) | 764 (36.7) |
| ≥30 | 240 (40.3) | 855 (39.7) |
| Physical activity, hours/week | **0.9 (0.1)** | **1.3 (0.1)** |
| Total cholesterol, mg/dL | 188.3 (2.7) | 194.1 (1.3) |
| Systolic blood pressure, mmHg | 131.0 (1.1) | 130.4 (0.5) |
| Digit Symbol Test | 50.8 (1.3) | 54.4 (0.6) |
| CERAD W-L delayed recall | 6.3 (0.2) | 6.4 (0.1) |
| CERAD W-L immediate recall | 19.9 (0.4) | 20.0 (0.2) |
| Animal Fluency | 18.4 (0.5) | 18.5 (0.2) |
